# From Silos to Synergy: Insights From Health Professionals on Integrating Youth Mental Health Care

**DOI:** 10.1111/eip.70213

**Published:** 2026-06-30

**Authors:** Vilas Sawrikar, Sarah Leung, Michael Hodgins, Catherine McHugh, Oliver Ardill‐Young, Valsamma Eapen, Raghu Lingam, Jackie Curtis

**Affiliations:** ^1^ Mindgardens Neuroscience Network Sydney New South Wales Australia; ^2^ Population Child Health Research University of New South Wales Sydney New South Wales Australia; ^3^ Growing Minds Australia, School of Psychology University of Sydney Sydney New South Wales Australia; ^4^ Discipline of Psychiatry and Mental Health University of New South Wales Sydney New South Wales Australia

**Keywords:** barriers and enablers, health professionals, integrated care, service integration, youth mental health care

## Abstract

**Background:**

Over the last two decades, integrated mental health services for youth aged 12–25 have expanded globally to enable young people to access comprehensive age‐appropriate care. This study examined the barriers and facilitators of integrated care from the perspective of health practitioners and service providers.

**Method:**

In total, 45 service managers and practitioners who work with young people aged 12–25 years participated in interviews investigating the barriers and enablers of integrated youth mental health care. Themes were identified through inductive analysis and organised by overarching themes.

**Results:**

Five overarching themes were identified: (i) *continuity of care*, referring to challenges associated with service fragmentation, navigation, and timely access to care; (ii) *workforce*, referring to challenges impacting staff ability to provide integrated care; (iii) *information exchange*, referring to areas to improve communication of patient information; (iv) *financing*, referring to supporting integrated care through core funding; and (v) *leadership*, referring to system‐ and service‐level priorities impacting service delivery.

**Conclusion:**

Improving integrated youth mental health care entails a multi‐level health system transformation. Strategies to achieve this are discussed in the context of the current results and emerging youth‐specific frameworks for strengthening service integration and integrated care.

Mental ill health is the leading health problem among young people worldwide, with mental disorders accounting for 45% of global burden of disease in youth aged 12–24 years (McGorry et al. 2024). Fifty percent of mental disorders first emerge before 15 years and 75% of mental illnesses develop by the age of 25 years, suggesting improving access to care for young people represents an important opportunity for early intervention and improving mental health outcomes over the life course (Solmi et al. 2022). To that end, governments around the world are increasing efforts to expand integrated youth mental health services based on research showing that integrated care is key to early intervention and treatment of mental disorders emerging in adolescence and young adulthood (McGorry et al. 2022; McHugh et al. 2024). However, current evaluation of youth mental health services preliminarily suggests significant challenges exist in delivering high quality integrated care (McGorry et al. 2022). The aim of this study was to understand the key barriers and facilitators of integrated youth mental health care from the perspective of health professionals to inform health system reforms toward delivering high quality early intervention for young people.

Gold‐standard models for mental health service delivery emphasise that young people's mental health needs are best met through multidisciplinary team‐based care. The needs of young people are expected to be met through input from several professionals, services and organisations (McGorry and Mei 2020). However, current reports on care experiences suggest that young people face difficulties accessing appropriate support due to high levels of service fragmentation, which perpetuates siloed rather than continuous, coordinated care across the youth mental health system (Hodgins et al. 2022). Young people and other stakeholders attribute these experiences to a lack of service integration due to factors such as individual services having high entry criteria, long wait lists, independent data management and communication systems, and separate funding and governance structures (MacDonald et al. 2021). Recently, we evaluated levels of integration in youth mental health service delivery in Australia to identify areas for improvement (Sawrikar, Hodgins, et al. 2025). The results highlighted the need to address structural aspects of service integration to reduce fragmentation.

Integrated models of mental health service delivery for youth aged 12–25 have expanded globally to address service fragmentation and enable young people to access age‐appropriate multidisciplinary care (McHugh et al. 2024). This includes introducing new models of primary and community‐based mental health services for young people, integrated social and community‐based care, digital platforms and interventions, and enhanced services for severe mental illnesses (McGorry et al. 2024; Colizzi et al. 2020; McGorry 2017; Rickwood et al. 2019). Integrated youth mental health care aims to provide timely access to health and social programs for mental health, substance use, physical health (including sexual health), and social care (including vocational and education support), typically relying on horizontal integration of services (Heeringa et al. 2020). At times, specialist mental health care may be required, involving referral to hospital, crisis services, or specialist clinicians, relying on vertical integration of services (Heeringa et al. 2020). Currently, there is no single model protocol operationalising integrated youth mental health service delivery, in part because implementation must account for local constraints (McGorry et al. 2022). In response, researchers, service providers and policymakers have presented guidelines for integrated service delivery, outlining clinical care pathways, essential elements of care and service values to ensure high‐quality service delivery within the youth mental health service context (Cross et al. 2019; Sawrikar, Buchan, and Gillespie‐Smith 2025; Sawrikar et al. 2021). These pathways present procedures for multidisciplinary care assessment, planning, coordination and review, alongside guidelines for triage and selecting care intensity, treatments, and duration of care.

Current research shows that effective delivery of integrated youth mental health care improves access to services, reduces treatment delays, and leads to better mental health outcomes for young people, making it key to high quality care (McHugh et al. 2024; Rickwood et al. 2019). To facilitate implementation, Hodgins et al. (Hodgins et al. 2022) recently developed the Youth Integration Project (YIP) framework as a youth mental health specific model for improving integration and strengthening integrated healthcare systems. YIP is comprised of the five building blocks of the World Health Organisation's health system framework: (1) Service delivery; (2) Workforce; (3) Information systems and communication; (4) Financing; and (5) Values, and leadership, governance and policy (Figure 1). The model proposes these represent the key components of integrated care, with each block comprising subcomponents for creating an integrated and continuous system of care for young people.

**FIGURE 1:**
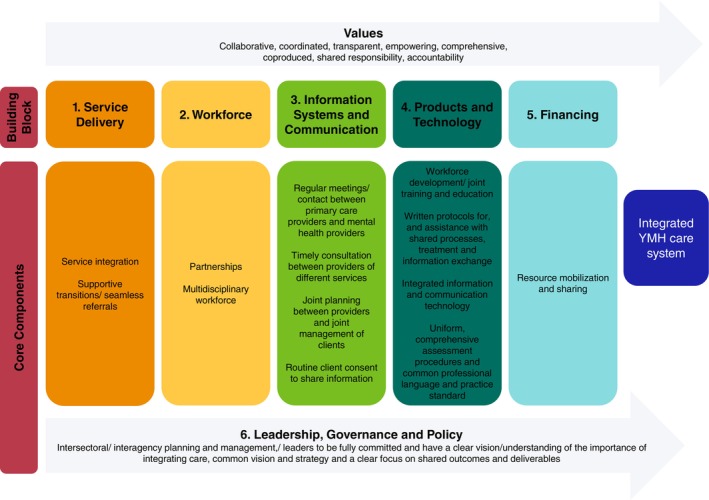
The key components of the Youth Integration Project (YIP) framework.

Empirical research into improving integrated healthcare systems has largely focused on understanding the barriers and facilitators of providing integrated service delivery to identify areas for improvement. Nooteboom et al. (Nooteboom et al. 2021) reviewed the facilitators and barriers professionals encounter when delivering integrated services for children and young people (0–18 years), noting that such delivery is shaped by a wide range of models designed to coordinate support across services (e.g., collaborative care, shared care). Their review highlighted the need to build workforce capacity, inter‐agency/inter‐professional collaboration and comprehensive needs‐led models of integrated service delivery. The findings are limited, since they are not specific to youth aged 12–25 years. Three qualitative studies have previously reported on professionals' experiences in establishing new integrated youth services underpinned by models of horizontal and vertical service integration (Nash et al. 2021; Chiodo et al. 2022; Callaly et al. 2011). Key barriers were competing models and agendas between services and staff perceptions of the negative impact of forming local partnerships. Key enablers were success in establishing strong, complementary partnerships with high stakeholder buy‐in, and shared resources. These results emphasise the salience of forming partnerships in establishing integrated services. However, the results are limited to experiences of working within new services and do not represent experiences of professionals across multiple services within a health network, which would extend knowledge beyond new service‐specific challenges and help identify areas to improve the sustainability of integrated care within a system.

In sum, the aim of the current study was to identify key barriers and facilitators that professionals have experienced in providing integrated youth mental health care within a local health system comprising primary, community, school, specialist and hospital‐based mental health services. The aim was to identify priority areas for improving integrated service delivery within a healthcare system comprising multiple providers across levels of care.

## 1 Methods

### 1.1 Study Design

A qualitative study using semi‐structured interviews, analysed through thematic analysis, was conducted to investigate health professionals' perspectives on the barriers and facilitators of providing integrated youth mental health care. Ethical approval was granted by the South Eastern Sydney Local Health District (SESLHD) ethics committee (2022/ETH02721). This study forms part of a larger study undertaken to understand service integration barriers, enablers, and solutions in a real‐world clinical environment.

### 1.2 Patient and Public Involvement

There was no patient or public involvement in the development of the study or preparation of this article.

### 1.3 Participants

Participants in this study included managers and practitioners who worked in services that delivered clinical care for mental health and related concerns for young people aged 12–25 years in SESLHD and Sydney Children's Hospital Randwick (SCHN). SESLHD services included the Bondi Junction Community Mental Health Centre; headspace Bondi Junction; and Prince of Wales Adolescent Service. SCHN services included the Saunders Unit; Emergency Department; and mental health inpatient and outpatient units. Participants were excluded from the study if they worked outside the geographic area of the study and/or worked exclusively with people younger than 12 or older than 25. We also excluded service providers from NGOs to focus on clinical services. In total, 45 service managers and practitioners (female = 37, male = 7, non‐binary = 1) consented and participated in the study. A purposive sample was used to represents front‐line providers who worked in services that delivered clinical care for mental health and related concerns for young people aged 12–25 years. This included 8 clinical team leaders, 5 school counsellors, 5 general practitioners, 2 paediatricians, 21 mental health clinicians (e.g., psychologists, psychiatrists, social workers, mental health nurses), and 4 peer workers.

### 1.4 Study Procedures

The study was advertised via organisational email distribution lists and personal links of the project steering committee, which involved executive stakeholders of SESLHD and SCHN. A REDCap link to the participant study information sheet and consent form (PISCF) was provided in the study advertisement. Participants who provided written consent to partake in the study were asked to submit details of a preferred method of contact. A research assistant contacted the consenting participants, and an interview time was arranged. Participants were not compensated for their time, as interviews were conducted during working hours and seen as contributing to their role.

Interviews were conducted by two interviewers (MH, SL) between March 2023 and August 2023. They were conducted either in person or via video on Microsoft Teams and were 30–60 min in duration. The lead interviewer (MH) has 10 years of experience conducting qualitative research in mental health services and provided training and supervision to the other interviewers (SL). Prior to starting the interviews, the interviewer explained the study to the participant and answered any questions the participant had. The interviews were semi‐structured, broadly following an interview guide (see Supporting Information) where questions prompted practitioners to report on barriers and facilitators of integrated care in line with the five components of the YIP framework (Hodgins et al. 2022). A reflexive approach was taken in which interview questions and prompts were continually adapted by the interviewer based on information obtained in previous interviews. Interviews were recorded and transcribed using the transcription software Otter (https://otter.ai/). Recordings were deleted after transcripts were complete. All identifiable information about participants (e.g., names, role, etc.) was removed and replaced with pseudonyms on transcripts. Data were stored securely on university servers.

### 1.5 Analysis Plan

De‐identified transcripts were coded in NVivo software using an inductive thematic approach (Braun and Clarke 2006). The analysis followed five key stages: (i) data familiarisation, (ii) generation of initial codes, (iii) searching for themes, (iv) grouping and reviewing themes, and (v) defining and naming themes. After becoming familiar with the data through multiple read‐throughs, initial codes of major themes and subthemes were developed inductively by three authors (ML, SL, VS). These codes were reviewed and further refined into a codebook through discussion between the authors. Since facilitator themes were the inverse of barrier themes, themes were coded into overarching themes and subthemes allowing for priority areas for improvement to be identified (Nooteboom et al. 2021). Results are presented in accordance with major thematic clusters and related barriers and facilitators for providers and practitioners to deliver integrated care as subthemes.

## 2 Results

### 2.1 Barrier and Enablers of Providing Integrated Care

Table 1 summarises the overarching themes of barriers and enablers that professionals experience in providing integrated care and includes direct quotes as examples of key issues. These were: continuity of care; workforce; information exchange; funding; and leadership.

**TABLE 1:** Key quotes supporting major themes and subthemes reported by practitioners in relation to the barriers and enablers of providing integrated care.

Overarching themes	Key issue	Barrier/problem	Enabler/solution
Continuity of care	Entry criteria	*Inconsistent application of service entry criteria policy* GP‐49: “I have worked here since 2019, I still can't quite get a hold of what our service will see or what candidates are appropriate” GP‐49: “Like who will be accepted into sort of which service? They'll say that it's written somewhere, it probably is. But on a practical level, it can change”	
		*Diagnosis‐related fragmentation* GP‐49: “One psychiatrist wouldn't see the young person about their gender dysphoria. But they would see them about their ADHD. So, then we're looking at one psychiatrist who would help with their depression and ADHD, and then a third sort of practitioner to help with the gender dysphoria”	
	Service gaps	*Gaps in services for youth with higher needs* GP‐45: “we're stuck with nowhere for these patients to go, you know, they're not bad enough that they go to public, but also too unwell for a lot of private psychiatrists”	
		*Unable to address client needs* SESLHD‐15: “I think there's a real mismatch between what people might perceive might be available and what is actually available when they come in”	
		*Private versus public healthcare split* SESLHD‐15: “it's just splintering of services due to privatisation and I think a lot of people scrambled for many years to fill in those gaps”	
	Wait times	*Long waitlist* P‐42: “People that are referred are waiting for up to a year or beyond who have really significant needs and the longer that they're not attended the more problematic and impact it has on their lives”	
	Reach	*Poor reach in the community* SESLHD‐10: “All I used to see was like, girls from Catholic schools with suicidal thoughts, you hardly got any kids from the public system, which to me says, you're not servicing your community”	*Outreach* SESLHD‐10: “People are just stuck in their little box, and they are just waiting for the patients to come to them, you know, it just doesn't work that way. I'd be going to community health centers. I'd be going to schools”
	Cost of care	*Out of pocket expenses* GP‐49: “A lot of services have out of pocket costs…within the ones that are free, generally it's pretty hard to get into with really long waitlist” SCHN‐52: “Most families are really struggling financially because one of the parents has to quit their job…. So we are struggling with knowing how to continue and how to support families who have children with special needs”	
		*Insufficient government subsidised sessions* GP‐49: “once you do, get into a service, there is a time cap and a lot of issues don't resolve in, say, the 10 session model that you have with Medicare. So you've got the situation that someone might access care, that's really good for a couple of sessions, but then there's no long‐term option” SC‐7: “I was in private practice when it was 20 and when it was 10 and just the difference that made, like a lot of our families are affluent, but there are still some that are here on a bursary.”	
Workforce	Lack of knowledge and confidence	*Low knowledge of services available* SESLHD‐15: “I think there's often vagueness partly because services change all the time” L‐20: “the mapping of all the services available to 12‐ to 25‐year‐olds, it's terribly complicated” SCHN‐81: “I think my concern is that we refer these young people to services we have no idea about. We could be sending them to somewhere that isn't very good, but we don't know.”	
	Limited staff capacity	*Unable to meet service demand* SESLHD‐15: “The fact that we only have two treating clinicians, and how this service gets like 10 new people every week, most of them are going to need a treating clinician—I think that's like fundamental problem with the model” SC‐01: “There are kids I see every week but I can't fit them all in. I'm already sitting here going I'm going to have to write so many emails to kids saying I'm sorry I couldn't see you this week.”	
		*Practitioner burden of multiple roles* SESLHD‐15: “My role has a lot of care coordination and casework. I'm the main point of contact with all the people and I'm doing a lot of referrals to other services.”	*Hire care coordinators* SESLHD‐23: “I would have a full‐time senior clinician and full‐time case managers…case managers make the world go round.”
			*Upskill low‐intensity workforce* L‐25: “I think what you need is a like a clinical nurse consultant, a mental health nursing workforce that is highly skilled and they can do other things while they're not doing mental health.”
	Staff turnover	*Loss of communication network* SESLHD‐15: “you lose a lot of knowledge in a sector, and you lose a lot of relationships that you build between agencies in order to have streamlined communication” SESLHD‐15: “The staff will turn over every 6 months. And so, it's impossible to have a cohesive like track for your young people because you don't have a cohesive like relationship, even between the staff”	
		Disruption in patient care SCHN‐81: “People have to move on, whatever, but sometimes the young people talk about the fact that they've got a relationship with someone and then that's had to stop”	
	Inter‐professional differences	*Different training pathways* SC‐01: “I feel like the education system actually contributes significantly to youth mental health, and I feel like if we worked together, we probably come up with something really, really amazing”	
Information exchange	Co‐location		*In‐person communication* SESLHD‐26: “Because they're [CASPAR] co‐located with the early psychosis team, it's not hard for a clinician to walk across and be like, hey, this is happening. What do you think about this person just makes it much more collegial and much more collaborative.” SESLHD‐26: “It's about having the sense that if there's other teams involved, you haven't just been abandoned with this patient” SESLHD‐26: “if someone's sitting in front of you, you just do develop relationships with them.”
	Information systems	*Different electronic medical record systems* P‐42: “We have electronic medical record which is not fit for purpose. It wasn't designed for community health and so our data collection is very slim”	
Funding	Community mental health service funding	*Short‐term or ad‐hoc funding of community/public health services* L‐20: “From a service manager perspective, it would help if youth mental health funding was ongoing, not all these temporary bits and pieces”.	*Invest in integration* L‐20: “The next improvement is in integrated care or integration across the board”
		Under resourcing of mental health services SESLHD‐10: “when you're referring to community services, they often not very well resourced at all. So that's where the gap is, is the resources available within community teams”	*Fund core mental health services* PW‐14: “I think investing a bunch of money into community mental health. Like if you could expand those services so that more people could be seen by them. That would be great”.
		*Different funding leading to service constraints* SESLHD‐15: “agencies funded by a particular government agency, do a particular job. For example, like youth homelessness services funded by the DCJ [Department of Community Justice] to do specifically that. That would mean that they have constraints on what and how they can work and the reporting they must do”	
Leadership	Prioritisation of service activity	*Preponderance to achieve targets of service activity* SESLHD‐10: “It seems like resources aren't used to improve services…it's basically just gathering statistics, trying to maintain levels of service”	
		*Service delivery overtaken by bureaucracy* SCHN‐58: “I think we're meant to be focused on advocating for the child… On the ground, it's not like that you know… it's been overtaken by bureaucracy… So I think actually what's good for the patient, what looks good on paper is not the same thing.”	
	Service policies	*Different service policies undermining consistency in clinical care* SESLHD‐15: “different organisations have different goals…. And there's sometimes, like ideological disagreements, I guess between services. Even just like gaps in systems where maybe people have different policies around confidentiality or consent”	
	Management	*Personal agendas undermining consistency in service policy* L‐20: “personalities of leaders in youth mental health leads to pushing certain agendas for one direction versus the other”.	

Abbreviations: GP, general practitioner; L, service leaders; P, paediatricians; PW, peer worker; SC, school counsellors; SCHN, Sydney Children Hospital Network; SESLHD, South Eastern Sydney Local Health District Clinicians.

### 2.2 Continuity of Care

Difficulty with providing continuity of care was identified as a major theme comprising five key issues: (i) *Entry criteria* referred to professionals' experience of inconsistent application of service entry criteria. This made it difficult to understand what services were available or who was eligible to receive services within their local health network. Services also had criteria for accepting or declining youth based on diagnosis leading to the need for multiple concurrent referrals to address the diverse youth mental health presentations; (ii) *Service gaps* referred to difficulties in finding appropriate care for youth with higher needs. The term “missing middle” was specifically used to describe clients who were considered to have needs too complex for primary mental health care services but did not meet the threshold of clinical severity that would indicate eligibility to state‐funded community mental health services; (iii) *Long wait times* referred to delays in accessing services. Wait times made it difficult for young people to gain timely access to care, which further placed them at risk for deterioration; (iv) *Reach* referred to having to operate within rigid models of in‐person care resulting in poor reach of mental health support in the community, especially reaching youth from low socioeconomic areas who do not readily come into clinical settings. Practitioners emphasised the salience of needing more outreach to be able to meet the mental health needs of young people in the community; and (v) *Cost of care* referred to barriers to accessing care due to high out of pocket expenses. Participants reported that there were an insufficient number of treatment sessions subsidised by the government to adequately address the needs of young people with longer term needs. Participants specifically referred to the recent change of reducing government subsidised sessions from 20 to 10 sessions for psychological therapy which limited continuity of care. Furthermore, practitioners working within hospital settings indicated that high costs of care were particularly problematic in families with youth with complex developmental needs due to the low availability of low‐cost service providers.

### 2.3 Workforce

Workforce‐related factors were identified as another major theme comprising four key issues: (i) *Lack of knowledge and confidence* referred to difficulties professionals experience in knowing what services were available. This problem was attributed to the siloing of services and treatment, making the healthcare system confusing to navigate. The lack of understanding created doubts about whether services were appropriate for clients; (ii) *Limited staff capacity* referred to difficulties in meeting the needs of their clients due to high workloads. This problem was highlighted by practitioners having to carry the dual role of delivering therapy and care coordination, which was suggested to be a suboptimal use of resources. Participants recognised the importance of care coordination but indicated a lack of staff with specialist knowledge of it. In enabling care coordination, two recommendations emerged: (a) *hire specialist staff for care coordination* whereby participants suggested that having a dedicated care coordinator within service teams would be an enabler of integrated care; and (b) *upskilling the low‐intensity workforce* whereby participants suggested that the gap in care coordination could be addressed by upskilling peer workers and school year coordinators to fulfil the role of care coordinator; (iii) *Staff turnover* was identified as a barrier to integrated care because of its impact on professionals maintaining links between services. Practitioners referred to their experiences of losing individual relationships with staff in other services, which they stated was key to maintaining referral networks and ensuring continuity of care for clients; and (iv) *Inter‐professional differences* referred to the need to overcome professional differences which created perceived artificial boundaries between settings.

### 2.4 Information Exchange

The way services exchange information was identified as a major theme comprising two key issues: (i) *Co‐location* was identified as an enabler of communication. Participants indicated that co‐location made it easier to have in‐person meetings, enabling effective communication between services. Practitioners also referred to the benefits of managing transfer of clinical responsibility. Finally, practitioners identified the benefits of developing professional relationships with practitioners from different services as a result of co‐location; and (ii) *Information systems* referred to the lack of centralised information systems as a barrier to providing integrated care. This was attributed to different data governance structures across settings whereby electronic medical records for patients were either hard to access or not fit for purpose in different healthcare settings.

### 2.5 Funding

Funding health services was identified as a major theme comprising one key issue: (i) *Community mental health services funding* for youth mental health was described as short‐term and ad‐hoc, making programs vulnerable to under‐resourcing, termination, and gaps in service provision. Furthermore, it was noted that there was a fragmentation in funding streams for agencies, which meant that agencies were constrained to only providing types of support funded. To address this, participants recommended allocating funding specifically to improve the availability of integrated care to a wider range of young people with complex needs. Practitioners also recommended consolidating funding into core mental health services rather than funding new short‐term programs in order to prevent under‐funding of health services.

### 2.6 Leadership

Service leadership was a major theme comprising three key issues: (i) *Prioritisation of service activity* referred to the perception of service managers giving higher priority to achieving activity targets rather than providing quality care. They stated there was a lack of values‐based decision‐making concerning the investment of resources into improving care for young people; (ii) *Service policies* referred to service agencies having separate service policies, which undermined practitioners' ability to work consistently with clients; and (iii) *Health leaders* referred to state and national leaders having personal agendas which determined the types of practices and policies that services adopted which ultimately influenced outcomes for service delivery.

## 3 Discussion

This study aimed to identify the key barriers and enablers that health professionals experience in providing integrated care with specific respect to the delivery of integrated youth mental health care within a community‐based and hospital health network. Barriers and enablers were organised by overarching themes of continuity of care, workforce, information exchange, financing, and leadership. As discussed below, the subthemes within each overarching theme help identify the key challenges health professionals experience in delivering integrated care and represent the priority areas for improving integrated youth mental health care.

The current findings suggest that improving integrated youth mental health care involves addressing practitioner‐, service‐, and system‐level barriers that impact integrated service delivery. The current results are consistent with existing literature suggesting the need to focus on service delivery models, workforce, inter‐service and inter‐professional collaboration and communication, financing, and leadership/management (Nooteboom et al. 2021; Chiodo et al. 2022; Callaly et al. 2011). However, the current results refine these specifically for sustained integration of services within a community‐based health network. For instance, our findings suggested that a key priority was addressing difficulties in providing continuity of care linked to problems with gaining service entry, rigid models of care, and service fragmentation, especially for youth with higher mental health needs. The specific lack of practitioners' understanding of continuous service availability, service entry criteria, and their own role in care coordination also reflected a lack of clear operationalisation of integrated service delivery within a healthcare system. Our results also reinforce previous findings that workforce capacity is a key barrier to integrated care (Callaly et al. 2011). Our results emphasised the salience of increasing burden on staff and fragmentation between disciplines, causing barriers to providing integrated care. Staff appear to be unable to focus on components of integrated care because of high service demand, inadequate resources, and lack of interdisciplinary collaboration. To that end, lack of leadership/policy on integrated care as well as insufficient funding to support staff appear key barriers as they seem to directly impact staff's knowledge and capability to facilitate integrated service delivery. In this study, fragmentation in governance and shortsighted leadership (i.e., activity‐focused goals and leadership influence) were cited as most problematic issues that needed immediate attention.

The enablers of integrated care identified in this study preliminarily clarify where financial support could be invested to enhance integrated service delivery. Our results support the need for greater provision of outreach services, i.e., delivering service in community‐based settings accessible to young people. The findings suggest equal consideration should be given to the integration of health, social, and community care in delivering integrated care and making care accessible (McHugh et al. 2024). To address issues of workforce, increasing workforce capacity to deliver care coordination was suggested, with our results suggesting options of recruiting care coordinators or upskilling the low‐intensity workforce. Finally, the current results are consistent with previous studies suggesting improving information exchange is a priority for providing integrated care, as it can address service fragmentation and help form and sustain partnerships (Nash et al. 2021; Callaly et al. 2011). Co‐location of services seems to be a potential solution, with multiple benefits of formalising shared care and interdisciplinary collaboration giving optimal continuity of care for patients (Nash et al. 2021).

### 3.1 Implications for Theory and Practice

The current perspective of health professionals suggests that integrated youth mental health care continues to be constrained by a lack of model clarity and system‐level limitations, undermining its long‐term sustainability. These constraints help explain the ongoing challenges and gaps identified across service structures and processes, as well as the burden on health professionals. System‐level factors related to a lack of resources, policy, leadership, and funding for integrated service delivery led to frontline practitioners having limited capability to provide the intended elements of integrated care. The implication from the current findings is to prioritise: (i) integrated service model development ensuring continuity of care, (ii) increasing workforce capacity, (iii) information exchange, (iv) integrated funding systems, and (v) leadership and governance mechanisms for promoting service integration. These domains converge with the YIP framework and are in keeping with key tenets of integrated care leading to recommendations for improvement based on existing literature (summarised in Table 2) (McHugh et al. 2024; Hodgins et al. 2022). First, we propose that service delivery should be guided by a unified protocol of stepped care that covers the complete spectrum of clinical need, as well as standardised intake and service entry procedures (Sawrikar, Hodgins, et al. 2025). Co‐location of services to enable communication may help to facilitate supportive transitions (Heyeres et al. 2016). It is also important to do regular service mapping exercises and disseminate this information, so providers are informed about the referral pathways that exist in local health areas (Price et al. 2019). Services may also broaden the scope of community outreach to increase accessibility of services. Second, investment should be considered for enhancing workforce capacity in relation to increasing the size of the workforce to meet service demand and diversifying roles to focus on care coordination. Workforce training will also be necessary in ensuring increased levels of knowledge of integrated care.

**TABLE 2:** Strategies for implementing the key components of service integration to address priorities for improvement.

Priority area	Component	Component attributes	Implementation strategies
Continuity of care	Service delivery	Service integration	Collaborative or stepped model of care; Community outreach; Service mapping to facilitate easier navigation and understanding of entry criteria
		Supportive transitions/seamless referrals	Shared care arrangements and referral pathways
Workforce development	Workforce	Partnerships	Increase knowledge and training in integrated services
		Multidisciplinary workforce	Systems of care coordination to access appropriate health professionals; Build capacity for care coordination; In depth appreciation of roles and culture
Forming and sustaining partnerships	Information systems and communication, products and technology	Regular meeting/contact	Co‐location to facilitate regular interservice clinical case review meetings; or in reach consultation arrangements; Facilitated ad‐hoc informal communication (email, phone) and case discussion
		Joint planning between providers and joint management of clients	Shared care arrangements; formal clinical case review meetings and progress updates
		Joint workforce development/joint training and education	Joint training activities
		Written protocols for shared processes, treatment, and information exchange	Standardised referral and discharge summaries
		Integrated information and communication technology	Facilitated informal ad hoc and email communication; Shared IT equipment
		Uniform assessment procedures	Joint assessments
	Finance	Resource mobilisation and sharing	Specialised shared care funding
	Leadership, governance, policy, and values	Interagency planning and management	Joint evaluation and strategy activities; service level agreements
		Commitment to shared vision	Attend business/operational/consortium meetings
		Shared values	Documented shared vision clearly communicated; Specify multiple shared values in bilateral agreement salient to delivery of youth mental health care delivery; Organisation leaders strongly support integration

Lastly, we propose issues of information exchange, funding, and leadership/governance should be the focus of forming and sustaining partnerships, which can be addressed using the last three components of YIP. Implementation should focus on two areas: (i) enabling regular meetings and ad hoc communication, centralised assessment and information exchange, and shared financing and resourcing for facilitating integrated service delivery; and (ii) establishing bilateral agreements that support shared care of clients between providers, especially in cases where separate governance structures exist. This could involve joint planning, staff training and leadership, governance, policy, and values. In a recent review, McHugh and colleagues showed the importance of these key elements of service integration. The most frequent components of youth mental health service integration were use of a multidisciplinary team, shared treatment planning and workforce training in the model, with shared electronic health records and regular team meetings also recognised for enabling interagency collaboration. These components of integration were associated with better clinical outcomes compared to treatment as usual (greater reduction in depressive symptoms) and improved treatment access and engagement. These findings emphasise the salience of service model and system‐level conditions in influencing young people's mental health outcomes and providing the right care.

### 3.2 Limitations and Future Directions

The following limitations of the current study should be noted to guide future research. While the current study focused on the views of service providers/practitioners, there is a need to understand the perspectives of young people and their families, as consumer and carer involvement in the development of service design is one of the main attributes of integrated care services. The current study is part of a larger project investigating barriers, enablers, and solutions of integrated youth mental health care with evaluation of consumers' perspectives underway. Furthermore, future research should also gather perspectives from diverse service providers and non‐government organisations, since integration of health and community services was identified as a priority for providing integrated care. We used thematic analysis to identify the key barriers and enablers of integrated care. However, this potentially limited our ability to explain how the barriers and enablers may be connected. The use of grounded theory approaches to construct a theory of change for improving integrated care may help to address this (Khan 2014). Finally, participants were recruited from one local health regional network in a well‐resourced urban area of Australia. Due to socioeconomic and demographic differences between local health districts, the generalisability of the findings may be limited to similar settings. There is a need to expand research into different regions to examine whether findings apply to other contexts.

## 4 Conclusion

The aim of the study was to examine the barriers and facilitators that practitioners experience in providing integrated youth mental health care to inform key priorities for improving integrated service delivery. The results suggested five main issues need to be addressed: ensuring continuity of care, workforce development in integrated care, facilitating information exchange, funding, and leadership and governance. We propose the emerging Youth Integration Project (YIP) framework has putative utility in strategically organising the key components of integrated care in a way that addresses these priority areas for improvement. Furthermore, future research needs to evaluate whether a health system transformation that targets these areas leads to improved care outcomes for young people. Evidence of positive outcomes provides support for a promising health systems approach to improving the quality of youth mental health care.

## Funding

This work was supported by the Australian Department of Health and Aged Care (no award/grant number).

## Ethics Statement

Study ethics was approved by the South Eastern Sydney Local Health District (SESLHD) Ethics Committee (reference numbers 2022/ETH02721).

## Consent

All participants provided written informed consent prior to enrolment in the study.

## Conflicts of Interest

The authors declare no conflicts of interest.

## Supporting information


**Data S1:** The Youth Integration Project.

## Data Availability

The data that support the findings of this study are available on request from the corresponding author. The data are not publicly available due to privacy and ethical restrictions.
