# Supplementary material for: From Silos to Synergy: Insights From Health Professionals on Integrating Youth Mental Health Care
Source: Early Interv Psychiatry. 2026 Jun 30;20(7):e70213. doi: 10.1111/eip.70213 (PMC13316723; doi:10.1111/eip.70213)
Supplement: Supplementary file 1 — Data S1: The Youth Integration Project. [file EIP-20-0-s001.docx]

**The Youth Integration Project**

**Clinician and manager Interview Guide**

***Introductions***

***Confirm consent and re-iterate that the interview will be recorded; transcripts will be de-identified and any quotes used will not be attributed by name***

**General**

Please describe your role and the service you work in

**Within-service - values**

How would you describe the values or vision of your service?

**Service delivery**

Can you describe what your service does? *Prompts: what population do you serve? What is the entry criteria? How strict are these criteria?*

What is the **intake and assessment** process like for your service? *How do clients come into your service?* *Prompts: Referrals? Screening? Soft entry/warm handover? Intake meetings? Walk in? Do you use standardised assessment tools or measures?*

What kind of information is made available to you about new patients? *How thorough is the assessment info. you receive? Is there room for improvement?*

How do you provide information to young people and carers? *What sort of tools/ modalities do you use? I.e. social media, paper-based, etc.*

Do you incorporate family engagement in your service delivery? *If so, how? How do you engage with carers? How often, for what reasons?*

***Ending process/ onward referrals***

How do clients come to end their time with your service? E.g., *they have fully recovered, they disengage, service is time limited, transitioned to adult*

*How/where do you refer clients to after finishing with your service? Difficulties?*

**Consumer and carer experience of care**

Are there any common causes of disruptions to care or frustrations for YP and families around delivery of care?

**Health workforce/ Training**

What other roles are present within your service? *Prompts: Remote or co-located? Training, background of other team roles? Blur and blend vs. distinct roles? Level of experience? Ie. How many years out of training? What is the proportion of new staff? Staff turnover? What kind of training does your team do? Details- how often? together/ individual, mandated/choice. Is training shared with other YMH/MH services in the same organisation or area?*

**Communication and Information systems**

How do you discuss clients within your team? Prompts: *Case reviews? Intake meetings? Corridor conversations? Email? Phone?*

How do you store and manage information within your service? *Prompts: Who can access this data? with other services?*

**Other services**

What other services do you work with on a regular basis? *Prompts: How do you work with them? How often? Why? Ie. ‘Shared care’ vs. transitioning/ referring clients onward*

How do you communicate with those services? *Share information? Do you develop shared treatment plans? How are they accessed/ updated by clinicians, the YP? Do you share treatment planning and assessments? How and when?*

How are these other services different to yours? *Do they manage different problems, populations? Do they have different intake criteria? How confident are you in understanding these? Vision?*

How do you think communication structures within and beyond your service could be improved?

**Governance and Finance**

What is the billing and funding model of your service?

Is this different to the other services that you mentioned above?

Does funding create any problems when you are referring a YP to other services? *Ie. out of pocket costs? Do YP or carers talk to you about this?*

How are you supported to work with other services?

**Summary questions**

If you could make one change to the current youth mental health model in the area, what would it be and why?

**The Youth Integration Project**

**GP/Counsellor Interview Guide**

***Introductions***

***Confirm consent and re-iterate that the interview will be recorded; transcripts will be de-identified and any quotes used will not be attributed by name***

**General**

1. Please describe your experience and the school or practice you work in.
2. Please describe the work you do in relation to youth mental health.
3. Are there any common causes of disruptions to care or frustrations for YP and families around accessing mental health services?
4. How do you discuss care of consumers within and beyond your team? Prompts: *Case reviews? Intake meetings? Corridor conversations? Email? Phone?*
5. How do you store and manage information about consumers? *Prompts: Who can access this data? with other services?*
6. What other services do you work with on a regular basis? *Prompts: How do you work with them? How often? Why? Ie. ‘Shared care’ vs. transitioning/ referring clients onward*
7. How do you communicate with those services? *Share information? Do you develop shared treatment plans? How are they accessed/ updated by clinicians, the YP?*
8. How do you think communication structures within and beyond your school/practice could be improved?
9. Does funding create any problems when you are referring a YP to other services? *Ie. out of pocket costs? Do YP or carers talk to you about this?*
10. What kind of reporting do you have to do? Frequency, to who?
11. How are you supported to work with other services?
12. If you could make one change to the current youth mental health model in the area, what would it be and why?
